# Mesenchymal stem cell–secreted extracellular vesicles carrying TGF‐β1 up‐regulate miR‐132 and promote mouse M2 macrophage polarization

**DOI:** 10.1111/jcmm.15860

**Published:** 2020-09-23

**Authors:** Yongqi Wang, Biao Han, Yingbin Wang, Chunai Wang, Hong Zhang, Jianjun Xue, Xiaoqing Wang, Tingting Niu, Zhen Niu, Yuhe Chen

**Affiliations:** ^1^ Department of Anesthesiology the First Hospital of Lanzhou University Lanzhou China; ^2^ Department of Thoracic Surgery the First Hospital of Lanzhou University Lanzhou China; ^3^ Department of Anesthesiology Lanzhou University Second Hospital Lanzhou China; ^4^ Department of Anesthesiology Gansu Provincial Hospital of TCM Lanzhou China; ^5^ The First School of Clinical Medicine the First Hospital of Lanzhou University Lanzhou China

**Keywords:** extracellular vesicles, M2 polarization, macrophages, mesenchymal stem cells, microRNA‐132, Mycbp2, TGF‐β1, TSC2

## Abstract

The effects of mesenchymal stem cells (MSCs) on different types of diseases are controversial, and the inner mechanisms remain unknown, which retards the utilization of MSCs in disease therapy. In this study, we aimed to elucidate the mechanisms of MSCs‐extracellular vesicles (EVs) carrying transforming growth factor‐beta 1 (TGF‐β1) in M2 polarization in mouse macrophages via the microRNA‐132 (miR‐132)/E3 ubiquitin ligase myc binding protein 2 (Mycbp2)/tuberous sclerosis complex 2 (TSC2) axis. Mouse MSCs were isolated for adipogenic and osteogenic induction, followed by co‐culture with mouse macrophages RAW264.7. Besides, mouse macrophages RAW264.7 were co‐cultured with MSCs‐EVs in vitro, where the proportion of macrophages and inflammation were detected by flow cytometry and ELISA. The experimental data revealed that MSCs‐EVs promoted M2 polarization of macrophages, and elevated interleukin (IL)‐10 expression and inhibited levels of IL‐1β, tumour necrosis factor (TNF)‐α and IL‐6. MSC‐EV‐treated macrophages RAW264.7 increased TGF‐β1 expression, thus elevating miR‐132 expression. MiR‐132 directly bound to Mycbp2, as confirmed by luciferase activity assay. Meanwhile, E3 ubiquitin ligase Mycbp2 could ubiquitinate TSC2 protein. Furthermore, silencing TGF‐β1 inhibited M2 polarization of MSC‐EV‐treated macrophages. Taken conjointly, this study provides evidence reporting that MSC‐secreted EVs carry TGF‐β1 to promote M2 polarization of macrophages via modulation of the miR‐132/Mycbp2/TSC2 axis.

## 1 INTRODUCTION

Macrophages are known as tissue‐resident or recruited cells, and they have essential functions in pathogen recognition, initiation of host defence through the protective inflammation.^1^ Two differentiation patterns, namely M1 and M2, have been characterized. M1 macrophages function as modulators of the host defence system, which are able to protect from infection because of protozoa, bacteria and viruses.^2^ M1 macrophages have a pro‐inflammatory effect and can produce inflammatory factors such as interleukin (IL)‐1β, tumour necrosis factor (TNF)‐α and IL‐6.^3^ M2 macrophages are responsible for tissue repair and reconstruction by secreting anti‐inflammatory cytokines that modulate cell replacement, angiogenesis and matrix remodelling.^4^ Macrophage polarization is emerged as a vital pathogenetic factor in neoplastic and inflammatory diseases.^5^ Polarization from M1 macrophages to M2 macrophages can reduce inflammatory responses and promote tissue repair and regeneration.^1^ Therefore, controlling excessive inflammatory response and maintaining a balance between pro‐inflammatory and anti‐inflammatory responses play a key role in promoting M2 polarization of macrophages.

Mesenchymal stem cells (MSCs) have become a hot issue as potent therapeutic tools for cell‐based therapy due to their characteristics of self‐renewal and differentiation into various tissues.^6^ According to previous literature,^7^, ^8^ MSCs can affect the polarization of macrophages in different diseases. MSCs‐extracellular vesicles (EVs) are secreted in large numbers, including both large micro‐vesicles and smaller exosomes with microRNAs (miRNAs), proteins as well as DNA that could modulate the gene expression along with functionality of recipient cells.^9^ Evidence has shown that MSCs‐EVs stimulated by lipopolysaccharide (LPS) can differentiate macrophages into a protective phenotype, thereby impacting cytokine secretion and enhancing phagocytic activity as well as inducing haematopoiesis and tissue repair.^10^ Besides being secreted by tumour educated‐stromal cells and carcinoma cells, transforming growth factor‐beta 1 (TGF‐β1) can also be produced by MSCs in the tumour environment.^11^ Meanwhile, a study has demonstrated that ^12^ TGF‐β1 can promote M2 polarization of macrophages. Li et al^13^ have found that miR‐132 expression could be up‐regulated by TGF‐β1, and miR‐132 induces M2 polarization in macrophages through regulating multiple transcription factors and adaptor proteins.^13^ E3 ubiquitin ligase myc binding protein 2 (Mycbp2) is a highly conserved protein that directly interacts with the transcriptional activation domain of myc.^14^ Mycbp2 has been proposed to possess the ability to ubiquitinate and degrade tuberous sclerosis complex 2 (TSC2) protein,^15^ and TSC2 protein can promote M2 polarization of macrophages.^16^ Since great achievements have been achieved in regulating the polarized activation of macrophages, the mechanisms remain to be further understood. Therefore, we conducted this study to investigate the mechanisms of MSCs‐EVs carrying TGF‐β1 in regulating M2 polarization of mouse macrophages via the miR‐132/Mycbp2/TSC2 axis.

## 2 MATERIALS AND METHODS

### 2.1 Cell culture and treatment

Mouse macrophages RAW264.7 (American Type Culture Collection) were thawed in a 37°C water bath box and then transferred to a 15‐mL centrifuge tube to remove the supernatant. The suspended cell suspension was moved to a 25 cm^2^ culture bottle and then incubated at 37°C with 5% CO_2_. The liquid was renewed every 2 days. Upon reaching 80%‐90% confluence (approximately 1 × 10^7^ cells), RAW264.7 cells were treated with 500 ng/mL LPS (strain O55:B5; Sigma‐Aldrich) for 24 hours. Afterwards, the supernatant of each group was taken, and the expression of inflammatory factors IL‐1β, TNF‐α, IL‐6 and IL‐10 was detected by enzyme‐linked immunosorbent assay (ELISA). 3‐(4,5‐dimethylthiazol‐2‐yl)‐2, 5‐diphenyltetrazolium bromide (MTT) assay was employed to detect the viability of cells following treatment. The cells in each group were inoculated into 96‐well plates at a density of 1 × 10^4^ cells/well, with eight parallel wells in each group. Additionally, a blank control well with only culture medium and no cells was set, and three time‐points: 24, 48 and 72 hours, respectively, were set, followed by subsequent experiments. When cells grew to 70% confluence, 5 mg/mL MTT solution (10 μL; ST316; Beyotime Institute of Biotechnology) was added to each well, and incubated in a 37°C incubator for 4 hours, followed by supernatant removal. After PBS washing, each well was added with 100 μL DMSO (D5879; Sigma) and incubated by shaking for 10 minutes. Thereafter, a microplate reader (MK3; Thermo) was applied to measure the optical density (OD) value of each well at 490 nm. Cell viability = (OD value of the experimental well − OD value of the blank well)/OD value of the blank well. The experiment was repeated three times, and the average value was taken.

RAW264.7 cells at the logarithmic growth phase were seeded into a 6‐well cell culture plate at a density of 4 × 10^5^ cells/well. When cell confluence reached 80%‐90%, the plasmids of different groups were connected to pLV‐Neo (Inovogen Tech. Co.). Each sequence was provided by Sigma‐Aldrich. After sequencing, the plasmid and pLV‐Neo were cotransfected into HEK293T cells, and the supernatant of the culture medium containing lentiviral particles was collected to infect RAW264.7 cells, and stably transfected cell lines were selected. The cells were treated with miR‐132 mimic, overexpression (oe)‐Mycbp2, oe‐TSC2 or their corresponding negative controls (NCs).

### 2.2 Culture and identification of MSCs

The well‐grown C57BL/6 mice were killed and soaked in alcohol for 10 minutes. The femur and tibia of the mice were taken in a sterile environment and then placed in Dulbecco's modified Eagle's medium (DMEM; Gibco by Life Technologies) after the leg meat was removed with instruments. Both ends of the femur and tibia were removed with clean and sterile scissors, and a syringe was utilized to flush bone marrow cells into a 15‐mL centrifuge tube with DMEM to discard the supernatant. Cells were resuspended in DMEM containing 10% foetal bovine serum (FBS; Biowest) and 100 U/mL penicillin‐streptomycin (Gibco by Life Technologies).

Mesenchymal stem cells at passage 3 were detached with trypsin (Gibco by Life Technologies) and suspended with phosphate buffered saline (PBS) to adjust the cell concentration to 1 × 10^6^ cells/mL. Cell suspension (200 μL) was sub‐packaged into Eppendorf (EP) tubes, added with 5 μL of different fluorescence‐labelled monoclonal antibodies (LA, CD11b, Sca‐1, CD105, CD34, CD45, CD31 and CD29) and incubated at 4°C for 15 minutes. After that, each tube was supplemented with 2 mL PBS and centrifuged at 1000 r/min for 5 minutes to discard the supernatant and unbound antibody. Subsequently, each tube was suspended with 400 μL of 0.01 mol/L PBS containing 0.5% paraformaldehyde and mixed well. The isotype control group was set with the fluorescence‐labelled IgG antibodies in the same colour, and the cells in each tube were detected by flow cytometer.

Mesenchymal stem cells at passage 3 were detached with trypsin to prepare single cell suspension. The cell concentration was altered to 1 × 10^5^ cell/mL and spread on a six‐well plate. When the cells reached 60%‐70% confluence, the supernatant was discarded. The experimental group was added with 2 mL osteogenic differentiation induction complete medium (MUBMX‐90021; Cyagen Biosciences) for induction. The control group was added with an equal amount of DMEM. The fresh medium was renewed in the experimental and the control groups every 3 days, for 2‐3 weeks. Next, each well was fixed with 2 mL of 4% neutral formaldehyde for 30 minutes and dyed with 1.5 mL alkaline phosphatase dye for 5 minutes, and observed under a microscope.

Mesenchymal stem cells at passage 3 were detached with trypsin to prepare for single cell suspension. The cell concentration was altered to 3 × 10^5^ cell/mL and spread on a six‐well plate. When the cells reached 80%‐90% confluence, the supernatant was discarded. The experimental group was added with 2 mL adipogenic differentiation medium A (MUBMX‐90031; Cyagen Biosciences) and then 2 mL adipogenic differentiation medium B. The induction was performed with alternate induction by medium A and B. Next, each well was fixed with 2 mL of 4% neutral formaldehyde for 30 minutes and dyed with 1.5 mL oil red O dye for 5 minutes, and observed under a microscope.

### 2.3 EVs extraction and identification

The well‐grown bone marrow MSCs were cultured overnight in serum‐free DMEM. When the cell confluence reached 80%‐90%, the supernatant was collected. The cells were centrifuged at 2000 *g* at 4°C for 20 minutes to remove the cell debris, and the obtained supernatant was centrifuged at 10 000 g at 4°C for 1 hour at high speed. After that, the precipitate was suspended and precipitated in serum‐free DMEM containing 25 mmol/L hydroxyethyl piperazine ethanesulfonic acid (pH = 7.4), and the high‐speed centrifugation was repeated again. The supernatant was discarded, and the precipitate was stored at −80°C for use.^17^

Identification of EVs by a transmission electron microscopy: 30 μL EVs were added dropwise on a copper net. One minute later, the liquid was dried from the side with filter paper. Next, the EVs were supplemented with 30 μL of phosphotungstic acid solution (pH = 6.8), counterstained at room temperature for 5 minutes and photographed under a transmission electron microscope.

Extracellular vesicles particles were dissolved in radioimmunoprecipitation assay (RIPA) buffer and quantitatively identified using a bicinchoninic acid (BCA) protein assay kit (Thermo Fisher Scientific). The antibodies for EV identification used in Western blot analysis were as follows: TSG101 (ab125011, 1:1000), CD63 (ab134045, 1:1000) and CD9 (ab92726, 1:2000) (from Abcam).^18^

Detection of the diameter of EVs by dynamic light scattering: Zetasizer Nano‐ZS90 instrument (Malvern) and the excitation light wavelength (λ = 532 nm) were used for experiments. Dilute EV samples were diluted with 0.15 mol/L NaCl to the appropriate optical signal detection level (1:50) for detection.

### 2.4 Co‐culture of MSCs and mouse macrophages

Mouse macrophages RAW264.7 were placed in the lower chamber of a 6‐transwell plate (Corning) at 2 × 10^6^ cells/well. MSCs or GW4869 (inhibitor of EV release)‐treated MSCs were tiled in the upper chamber (0.4 μm pore size membrane) at 4 × 10^5^. After incubation, the culture supernatant and macrophages were collected for further experiments. The cells and supernatant were harvested and stored at 80°C until further use.

### 2.5 Co‐culture of MSC‐derived EVs and mouse macrophages

Extracellular vesicles from MSCs (1 μg EVs were dissolved in 100 μL PBS) ^19^ were labelled with PKH67 (green) staining solution (MINI67‐1KT; Sigma‐Aldrich). Next, EVs were co‐incubated for 48 hours with RAW264.7 cell culture supernatant that had been seeded in a 24‐well plate with 50%‐60% confluence and then stained with 4',6‐diamidino‐2‐phenylindole (DAPI) to observe nuclear morphology. The absorption of EVs by RAW264.7 cells was subsequently observed under a fluorescence microscope.

MSC‐derived EVs of different treatments were then co‐cultured with RAW264.7 cells: RAW264.7 group, RAW264.7 + MSCs‐EVs group, RAW264.7 + MSCs‐EVs si‐NC group and RAW264.7 + MSCs‐EVs si‐TGF‐β1 group. Expression of TGF‐β1 was determined by reverse transcription quantitative polymerase chain reaction (RT‐qPCR) and Western blot analysis.

### 2.6 RT‐qPCR

TRIzol reagent (Invitrogen) was used to extract the total RNA from the tissues or cells according to the instructions, and the RNA concentration was then determined. The primers used in this study were synthesized by Takara (Table 1). For miRNAs, polyA‐tailed detection kit (B532451; Sangon Biotech) was used to obtain cDNA of the polyA‐tailed miRNA (containing universal PCR primers reverse (R) and U6 universal PCR primers R). Non‐miRNA reverse transcription was performed in the light of the instructions of cDNA reverse transcription kit (K1622; Beijing Reanta Biotechnology Co., Ltd.). Detection was performed in a real‐time PCR instrument (ViiA 7; Da'an Gene Co., Ltd.). GAPDH was used as an internal reference primer to calculate the relative transcription level of the target gene using a relative quantitative method ($2^{‐\Delta \Delta C_{t}}$ method).^20^

**TABLE 1:** Primer sequences for RT‐qPCR

Gene	Sequence
miR‐132	5′‐TTAACAGTCTACAGCCATCCTCG‐3′
Mycbp2	F: 5′‐GCAAGGGATATCTGCAGTTGGACACC‐3′
	R: 5′‐GGAACCTCGAGTAGCCATATTGGCTAGC‐3′
TSC2	F: 5′‐GCAGCAGGTCTGCAGTGAAT‐3′
	R: 5′‐GCAGCAGGTCTGCAGTGAAT‐3′
TGF‐β1	F: 5′‐TGG TGG ACCGCAACAAC‐3′
	R: 5′‐AGCCACTCAGGC GTATCAG‐3′
U6	F: 5′‐TCCGACGCCGCCATCTCTA‐3′
	R: 5′‐TATCGCACATTAAGCCTCTA‐3′
GAPDH	F: 5′‐AACGACCCCTTCATTGAC‐3′
	R: 5′‐TCCACGACATACTCAGCAC‐3′

Abbreviations: F, forward; GAPDH, glyceraldehyde phosphate dehydrogenase; miR‐132, microRNA‐132; R, reverse; RT‐qPCR, reverse transcription quantitative polymerase chain reaction; TGF‐β1, transforming growth factor‐beta 1; TSC2, tuberous sclerosis complex 2.

### 2.7 Western blot analysis

Total protein was extracted from tissues or cells using high‐efficiency RIPA lysis buffer (C0481; Sigma‐Aldrich) following the instructions. The supernatant was extracted, and the protein concentration of each sample was determined using a BCA kit (23227; Thermo). The protein was quantified according to different concentrations. After separation by polyacrylamide gel electrophoresis, the protein was transferred to a polyvinylidene fluoride membrane, which was then blocked with 5% bovine serum albumin at room temperature for 1 hour. Thereafter, the membrane was probed with primary antibodies against inducible nitric oxide synthase (iNOS; 1:100, ab15323), CD86 (1:100, ab112490), CD206 (1:5000, ab125028), arginase‐1 (Arg‐1; 1:100, ab91279), TGF‐β1 (1:200, ab92486), TSC2 (1:1000, ab166790), GAPDH (1:5000, ab8245) (from Abcam) and Mycbp2 (1:2000, 12022‐1‐AP; ProteinTech Group). The next day, the membrane was re‐probed with horseradish peroxidase–labelled goat anti‐rabbit IgG (1:20 000, ab205718; Abcam) for 1.5 hours and then supplemented with developer (NCI4106; Pierce). ImageJ 1.48u software (Bio‐Rad) was used for quantitative protein analysis. The protein expression was analysed by the ratio of grey values of target band to that of the internal reference.

### 2.8 Flow cytometry

The cells were made into single cell suspension and resuspended in staining buffer (BD Biosciences). The cells were stained with F4/80 (eBioscience, 17‐4801‐82, rat, 1:50), CD86 (eBioscience, 25‐0862‐82, rat, 1:400) and CD206 (eBioscience, 12‐2061‐82, rat, 1:800), and then detected by a BD FACS Canto II flow cytometer (BD Immunocytometry Systems), and analysed by the Flowjo software.^21^

### 2.9 ELISA

The collected cell supernatant was used to detect the contents of inflammatory factors according to the instructions of IL‐1β, TNF‐α, IL‐6 and IL‐10 (MLB00C, MTA00B, D6050 and DY417‐05; R&D Systems) ELISA kits. The antigen was diluted with coated diluent at a ratio of 1:20, and each well was added with 100 μL standard diluent and left to react overnight at 4°C. The diluted sample was added into the reaction well of enzyme plate (100 μL per well). Negative and positive controls were set, and the duplicate well test was performed. Each well was added with 100 μL of enzyme conjugates diluted with sample diluent and reacted at 37°C for 30 minutes. Next, 100 μL of horseradish peroxidase substrate solution was added to the well and developed at 37°C for 10‐20 minutes without light exposure. When the positive control had obvious colour change or the NC had slight colour change, 50 μL of termination solution was added into each well to halt the reaction. After 20 minutes, a microplate reader (Synergy 2; BioTek) was used to measure the absorbance (A) value of each well at 450 nm, and the blank control well was adjusted to zero to measure the OD value of each well.

### 2.10 Dual‐luciferase reporter gene assay

The dual‐luciferase reporter gene vector of the target gene Mycbp2 3′untranslated region (UTR) and the mutants with miR‐132 binding site were constructed: PGLO‐Mycbp2‐wild type (WT) and PGLO‐Mycbp2‐mutant type (MUT). The two reporter plasmids were cotransfected into 293T cells with miR‐132 mimic plasmids and mimic‐NC plasmids. The cells were lysed 24 hours after transfection and centrifuged at 12 000 rpm for 1 minute to collect the supernatant. Dual‐Luciferase Reporter Assay System (E1910; Promega) was used to detect luciferase activity. Each sample was supplemented with 100 μL working solution of firefly luciferase and 100 μL working solution of renilla luciferase. The relative luciferase activity was calculated with the ratio of firefly luciferase activity to renilla luciferase activity.

### 2.11 Co‐immunoprecipitation (Co‐IP) assay

Transfected cells were lysed in lysis buffer (50 mmol/L Tris‐HCl, pH = 7.4; 150 mmol/L NaCl, 10% glycerol, 1 mmol/L ethylenediaminetetraacetic acid, 0.5% NP‐40 and a protease inhibitor mixture), and the cell debris was removed by centrifugation. The cleared cell lysate was incubated with anti‐HA or anti‐FLAG antibody (Sigma‐Aldrich Chemical Company) and 15Si protein A/G beads (Santa Cruz Biotechnology) for 2 hours. The lysate was then centrifuged at 3000 rpm and 4°C for 3 minutes to enable agarose beads to the bottom of the tube, with the supernatant removed. The complex of HES5 and antigen antibody at the bottom of the tube was subsequently collected and quantified. The agarose beads were washed with 1 mL of lysis buffer for three to four times and added with 15 μL of 2 × SDS sample buffer. After extensive washing, the beads were boiled at 100°C for 5 minutes. After denaturation, proteins were separated by sodium lauryl sulphate‐polyacrylamide gel electrophoresis, transferred to a nitrocellulose membrane (Millipore) and then immunoblotted.^22^

### 2.12 Statistical analysis

The data were processed using SPSS 19.0 statistical software (IBM Corp.). Measurement data were expressed as mean ± SD. Comparison between two groups was conducted using unpaired *t* test, while comparisons among multiple groups were conducted by one‐way analysis of variance (ANOVA) with Tukey's post hoc test. The data at different time‐points were compared by two‐factor ANOVA followed by Dunnett's correction. A value of *P* < .05 indicated significant difference.

## 3 RESULTS

### 3.1 Isolation and identification of MSCs

**FIGURE 1:**
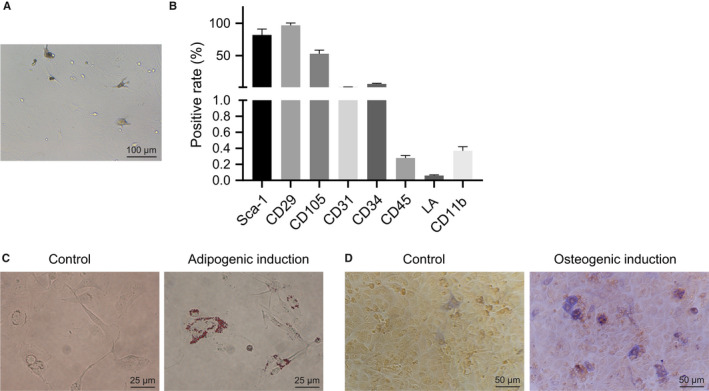
Isolation and identification of MSCs. A, MSCs at passage 3 were observed under an inverted microscope. B, Quantitative analysis of expression of MSC surface markers by flow cytometry. C, Oil red O staining analysis after adipogenic induction in MSCs. D, Alkaline phosphatase staining analysis after osteogenesis induction in MSCs. The data of the two groups were analysed by unpaired *t* test. Data are shown as mean ± SD of three technical replicates

The isolated and cultured MSCs were uniformly fibrous, long spindle‐shaped and vortex‐shaped (Figure 1A). Flow cytometric analysis showed that the cells overexpressed Sca‐1, CD29 and CD105, but they did not express or poorly expressed CD31, CD34, CD45, LA and CD11b (Figure 1B). Oil red O staining results suggested that a large number of red lipid droplets appeared after isolation of the cultured MSCs (Figure 1C). After osteogenesis induction, alkaline phosphatase activity was increased (Figure 1D). These results indicate the successful isolation of MSCs.

### 3.2 MSCs promote M2 polarization of LPS‐treated macrophages through EVs in vitro

**FIGURE 2:**
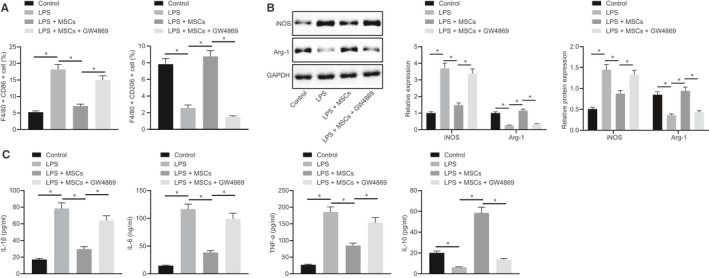
EVs from MSCs promote M2 polarization of macrophages. A, Quantitative analysis of cell ratio of CD86^+^ and CD206^+^ in F4/80 cells by flow cytometry in the co‐culture system of macrophages with MSCs or GW4869‐treated MSCs. B, Representative Western blots of iNOS and Arg‐1 proteins and their quantitation in the co‐culture system of macrophages with MSCs or GW4869‐treated MSCs, normalized to GAPDH. C, Expression of inflammatory factors was measured by ELISA in cell supernatant in the co‐culture system of macrophages with MSCs or GW4869‐treated MSCs. The data were analysed by one‐way ANOVA with Tukey's post hoc test. Data are shown as mean ± SD of three technical replicates. *, *P* value between groups was less than 0.05

With the aim to probe into the effect of MSCs and EVs on mouse macrophage polarization, we co‐cultured RAW264.7 cells with MSCs or GW4869‐treated MSCs. Flow cytometry was used to detect the M1 macrophage surface marker CD86 and M2 macrophage surface marker CD206. The results (Figure 2A) displayed that CD86^+^ cells were increased, while CD206^+^ cells were decreased in RAW264.7 cells after LPS treatment. The proportion of CD86^+^ cells was decreased, while the proportion of CD206^+^ cells was increased in RAW264.7 cells co‐cultured with LPS‐treated MSCs, which was negated by GW4869 treatment. Cell viability measured by MTT assay showed no difference in RAW264.7 cells following LPS treatment or without any treatment (Figure S1). Subsequently, RT‐qPCR and Western blot analysis were conducted to detect the expression of M1 macrophage marker iNOS and M2 macrophage marker Arg‐1 in macrophages, and the results showed (Figure 2B) reduced iNOS and elevated Arg‐1 in the co‐culture system of RAW264.7 cells with MSCs, while the decrease in iNOS and increase in Arg‐1 were reversed after co‐culture of GW4869‐treated MSCs and RAW264.7 cells. Next, the expression of IL‐1β, IL‐6, TNF‐α and IL‐10 in the cell supernatant was detected by ELISA. The results indicated (Figure 2C) that the expression of IL‐1β, IL‐6 and TNF‐α was increased while that of IL‐10 was decreased in RAW264.7 cells after LPS treatment, which was reversed in RAW264.7 cells co‐cultured with MSCs. After inhibiting the secretion of EVs, the expression of inflammatory factors was reversed in RAW264.7 cells. The above results indicate that EVs from MSCs promote M2 polarization of macrophages in vitro.

### 3.3 TGF‐β1 in MSC‐derived EVs promotes miR‐132 expression in macrophages and thus promotes M2 polarization

**FIGURE 3:**
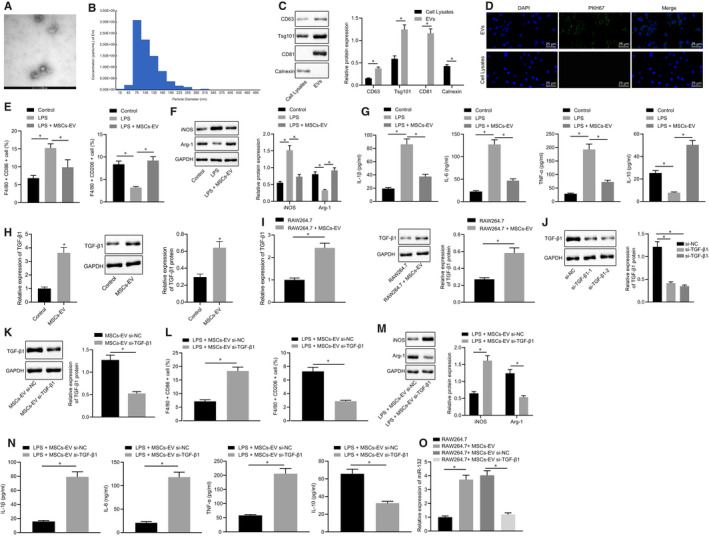
MSCs‐EVs deliver TGF‐β1 to promote miR‐132 expression in macrophages and thus promote M2 polarization of mouse macrophages. A, Identification of EVs by transmission electron microscopy (20 000×). B, Detection of EVs diameter by dynamic light scattering. C, Representative Western blots of CD9, CD63 and TSG101 proteins and their quantitation in cell lysate, normalized to GAPDH. D, Observation of EV uptake by macrophage cells under an inverted fluorescence microscope (400×). E, Quantitative analysis of proportion of CD86^+^ and CD206^+^ in F4/80 cells by flow cytometry in the co‐culture system of RAW264.7 cells with MSCs‐EVs. F, Representative Western blots of iNOS and Arg‐1 proteins and their quantitation in the co‐culture system of RAW264.7 cells with MSCs‐EVs, normalized to GAPDH. G, Expression of inflammatory factors was measured by ELISA in cell supernatant in the co‐culture system of RAW264.7 cells with MSCs‐EVs. H, TGF‐β1 expression was determined by RT‐qPCR and Western blot analysis in MSC‐derived EVs, normalized to GAPDH. I, TGF‐β1 expression was determined by RT‐qPCR and Western blot analysis in the co‐culture system of RAW264.7 cells with MSCs‐EVs, normalized to GAPDH. J, Representative Western blots of TGF‐β1 protein and its quantitation in MSCs‐EVs treated with si‐TGF‐β1‐1 or si‐TGF‐β1‐2, normalized to GAPDH. K, Representative Western blots of TGF‐β1 protein and its quantitation in the co‐culture system of RAW264.7 cells with si‐TGF‐β1‐treated MSCs‐EVs, normalized to GAPDH. L, Quantitative analysis of proportion of CD86^+^ and CD206^+^ in F4/80 cells by flow cytometry in the co‐culture system of RAW264.7 cells with si‐TGF‐β1‐treated MSCs‐EVs. M, Representative Western blots of iNOS and Arg‐1 proteins and their quantitation in the co‐culture system of RAW264.7 cells with si‐TGF‐β1‐treated MSCs‐EVs, normalized to GAPDH. N, Expression of inflammatory factors was measured by ELISA in cell supernatant upon co‐culture of RAW264.7 cells with si‐TGF‐β1‐treated MSCs‐EVs. O, miR‐132 expression was determined by RT‐qPCR in the co‐culture system of RAW264.7 cells with si‐TGF‐β1‐treated MSCs‐EVs, normalized to U6. The data of the two groups were analysed by unpaired *t* test, while the data of multiple groups were analysed by one‐way ANOVA with Tukey's post hoc test. Data are shown as mean ± SD of three technical replicates. *, *P* value between groups was less than 0.05

Next, we used ultra‐high‐speed centrifugation to extract EVs from MSCs. EVs observed under a transmission electron microscopy (Figure 3A) presented round or oval membrane vesicles with basically the same shape. EVs range in size from 30 to 120 nm by dynamic light scattering (Figure 3B). As shown in Figure 3C, EVs highly expressed positive markers CD63, CD81 and TSG101, but did not express negative marker calnexin, confirming the successful extraction of EVs (*P* < .05). In order to elucidate whether macrophages could absorb EVs of MSCs, we co‐cultured PKH67 (green)‐labelled EVs with macrophages RAW264.7 in vitro and co‐cultured for 12 hours, and then, uptake of EVs was observed under a confocal fluorescence microscope. The findings suggested that RAW264.7 cells exhibited green fluorescence when compared to EV‐depleted cell lysate, indicating that RAW264.7 cells could take up PKH67‐EVs, and that EVs could be transferred from MSCs to macrophages RAW264.7 (Figure 3D). For verifying the effect of MSCs‐EVs on macrophage polarization in vitro, LPS‐treated RAW264.7 cells were co‐cultured with MSCs‐EVs, and then the M1 macrophage surface markers CD86 and M2 macrophage CD206 were detected. The results illustrated that (Figure 3E) CD86^+^ cells were increased and CD206^+^ cells were decreased after LPS treatment. The proportion of CD86^+^ was decreased, while the proportion of CD206^+^ was increased in the co‐culture system of LPS‐treated RAW264.7 cells with MSCs‐EVs in comparison to LPS‐treated RAW264.7 cells without co‐culture with MSCs‐EVs. Subsequently, RT‐qPCR and Western blot analysis were conducted to detect the expression of M1 macrophage marker iNOS and M2 macrophage marker Arg‐1 in RAW264.7 cells, and the results showed (Figure 3F) reduced iNOS and elevated Arg‐1 in RAW264.7 cells co‐cultured with MSCs‐EVs. Next, the expression of IL‐1β, IL‐6, TNF‐α and IL‐10 was detected by ELISA in the cell supernatant. The results indicated (Figure 3G) that the expression of IL‐1β, IL‐6 and TNF‐α was increased, while IL‐10 expression was decreased in RAW264.7 cells after LPS treatment, which was undermined following co‐culture with MSCs‐EVs. The above results indicate that MSCs‐EVs can promote M2 polarization of macrophages in vitro.

TGF‐β1 has been shown to be activated by MSCs‐EVs in lung cancer cells, while silencing TGF‐β1 expression in MSCs can enhance the anti‐proliferative and pro‐apoptotic effects of MSCs on lung cancer cells via MSCs‐EVs.^11^ Moreover, TGF‐β1 could promote macrophages polarized to M2 phenotype in mice.^12^ A previous study revealed that TGF‐β1 can promote the expression of miR‐132.^13^ Here, we first determined the expression of TGF‐β1 by RT‐qPCR and Western blot analysis in MSCs‐EVs, which revealed an enhancement in the expression of TGF‐β1 in MSCs‐EVs at the transcription and protein levels (Figure 3H). In order to study whether MSCs‐EVs could carry TGF‐β1 into macrophages and thereby promote M2 polarization of macrophages, we determined TGF‐β1 expression in RAW264.7 cells co‐cultured with or without MSCs‐EVs by Western blot analysis (Figure 3I). RAW264.7 cells with MSCs‐EVs presented increased protein expression of TGF‐β1. This result indicates that MSCs‐EVs can carry TGF‐β1 into macrophages. To further find out the role of TGF‐β1 in this process, we knocked TGF‐β1 down in MSCs‐EVs and examined its expression in MSCs‐EVs (Figure 3J). The results showed that si‐TGF‐β1‐1 and si‐TGF‐β1‐2 reduced the expression of TGF‐β1 in MSCs‐EVs, of which si‐TGF‐β1‐2 was more efficient, and thus selected for subsequent experiments. Afterwards, RAW264.7 cells were treated with EVs with TGF‐β1 silencing, and the results showed (Figure 3K) that the expression of TGF‐β1 was reduced in RAW264.7 cells co‐cultured with MSCs‐EVs treated with si‐TGF‐β1. Further experimental results presented increased CD86^+^ cells, decreased CD206^+^ cells, elevated iNOS expression and reduced Arg‐1 expression, as well as elevated IL‐1β, IL‐6 and TNF‐α expressions while decreased IL‐10 expression upon treatment of MSCs‐EVs with si‐TGF‐β1 (Figure 3L‐N). The above results indicate that silencing TGF‐β1 in MSCs‐EVs can inhibit M2 polarization of macrophages in vitro.

The next step was to investigate whether MSCs‐EVs could carry TGF‐β1 to promote the expression of miR‐132 in macrophage RAW264.7. The expression of miR‐132 determined using RT‐qPCR (Figure 3O) was found to be enhanced in RAW264.7 cells co‐cultured with MSCs‐EVs, which was decreased in RAW264.7 cells co‐cultured with MSCs‐EVs treated with si‐TGF‐β1. This result indicates that MSCs‐EVs carry TGF‐β1 to promote miR‐132 expression in RAW264.7 cells.

### 3.4 miR‐132 targets Mycbp2 in macrophages

**FIGURE 4:**
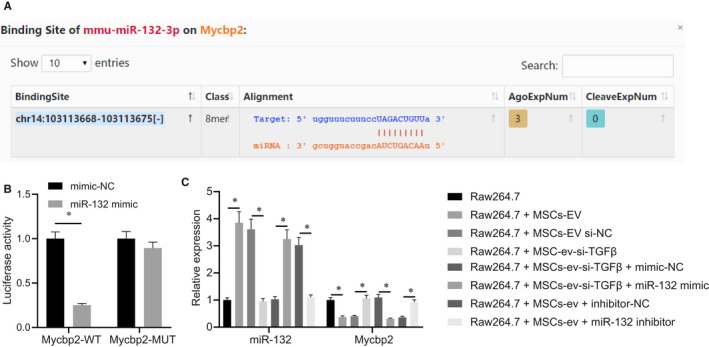
miR‐132 targets Mycbp2 in RAW264.7 cells. A, Putative miR‐132 binding sites in the 3’UTR of Mycbp2 mRNA in the online prediction software starbase. B, Binding of miR‐132 to Mycbp2 confirmed by dual‐luciferase reporter gene assay in 293T cells. C, miR‐132 expression and Mycbp2 mRNA expression were determined by RT‐qPCR in the co‐culture system of RAW264.7 cells with MSCs‐EVs treated with si‐TGF‐β1, si‐TGF‐β1 + miR‐132 mimic or miR‐132 inhibitor, normalized to U6 and GAPDH, respectively. The data of the two groups were analysed by unpaired *t* test, while the data of multiple groups were analysed by one‐way ANOVA with Tukey's post hoc test. Data are shown as mean ± SD of three technical replicates. *, *P* value between groups was less than 0.05

In order to further elucidate the downstream mechanism of miR‐132 in regulating macrophage differentiation, we predicted through an online prediction software starbase that miR‐132 could target Mycbp2 (Figure 4A). Meanwhile, the results of the dual‐luciferase reporter gene assay (Figure 4B) revealed that the luciferase activity of Mycbp2‐WT was decreased in cells transfected with miR‐132 mimic, indicating that miR‐132 could specifically bind to the 3'UTR of Mycbp2 mRNA. We further inhibited the expression of miR‐132 in macrophages, and the expression of Mycbp2 was detected using RT‐qPCR in RAW264.7 cells. As shown in Figure 4C, Mycbp2 mRNA expression was reduced in the co‐culture system of RAW264.7 cells with MSCs‐EVs or with si‐NC‐treated MSCs‐EVs, while it was increased upon si‐TGF‐β1 treatment. Furthermore, combined treatment with si‐TGF‐β1 and miR‐132 mimic resulted in a decline in the Mycbp2 mRNA expression in the co‐culture system of RAW264.7 cells with MSCs‐EVs, whereas miR‐132 inhibitor treatment brought about opposite results (*P* < .05). Altogether, miR‐132 can target Mycbp2 and negatively regulate its expression in RAW264.7 cells.

### 3.5 miR‐132 promotes M2 polarization by targeting Mycbp2 in macrophages

**FIGURE 5:**
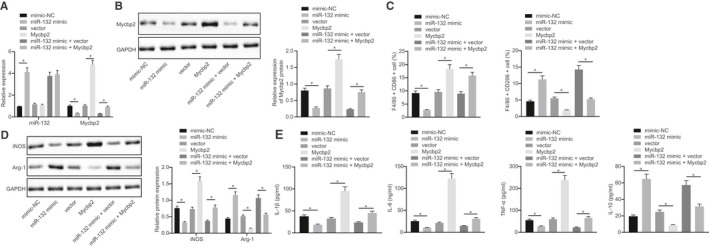
miR‐132 targets Mycbp2 in macrophages to promote M2 polarization of macrophages. A, miR‐132 expression and Mycbp2 mRNA expression were determined by RT‐qPCR in RAW264.7 cells treated with miR‐132 mimic, Mycbp2 or both, normalized to U6 and GAPDH, respectively. B, Representative Western blots of Mycbp2 protein and its quantitation in RAW264.7 cells treated with miR‐132 mimic, Mycbp2 or both, normalized to GAPDH. C, Quantitative analysis of cell ratio of CD86^+^ and CD206^+^ in F4/80 cells by flow cytometry in the presence of miR‐132 mimic, Mycbp2 or both. D, Representative Western blots of iNOS and Arg‐1 proteins and their quantitation, normalized to GAPDH. E, Expression of inflammatory factors was measured by ELISA in cell supernatant in the presence of miR‐132 mimic, Mycbp2 or both. The data of multiple groups were analysed by one‐way ANOVA with Tukey's post hoc test. Data are shown as mean ± SD of three technical replicates. *, *P* value between groups was less than 0.05

As described above, MSCs‐EVs could carry TGF‐β1 to promote the expression of miR‐132 and promote M2 polarization, and meanwhile, miR‐132 targeted Mycbp2. In order to further figure out the effect of miR‐132 regulating Mycbp2 on macrophage polarization, we inhibited miR‐132 and Mycbp2 in macrophages. After LPS treatment, miR‐132 expression and Mycbp2 mRNA expression were determined by RT‐qPCR and Mycbp2 protein expression by Western blot analysis (Figure 5A,B). The obtained results suggested that miR‐132 expression was elevated and Mycbp2 expression was decreased in cells with miR‐132 mimic, and Mycbp2 expression was increased in cells with overexpressed Mycbp2 along with both miR‐132 mimic and overexpressed Mycbp2.

Subsequently, a series of assay were performed to further confirm the roles of miR‐132 and Mycbp2 in M2 polarization of macrophages. The further experiments exhibited decreased CD86^+^ cells, elevated CD206^+^ cells, reduced iNOS expression and enhanced Arg‐1 expression, as well as decreased IL‐1β, IL‐6 and TNF‐α expressions while increased IL‐10 expression in RAW264.7 cells overexpressing miR‐132. On the contrary, the tendencies were opposite in RAW264.7 cells with overexpressed Mycbp2. Except that, we also found that overexpression of Mycbp2 reversed the effects of overexpressed miR‐132 on M2 polarization of macrophages (Figure 5C‐E). The above results indicate that miR‐132 can target Mycbp2 in macrophages to promote M2 polarization of macrophages.

### 3.6 MSC‐derived EVs carrying TGF‐β1 elevate miR‐132 expression and down‐regulate MYCBP2 expression, polarizing macrophages towards M2 phenotype in vitro

**FIGURE 6:**
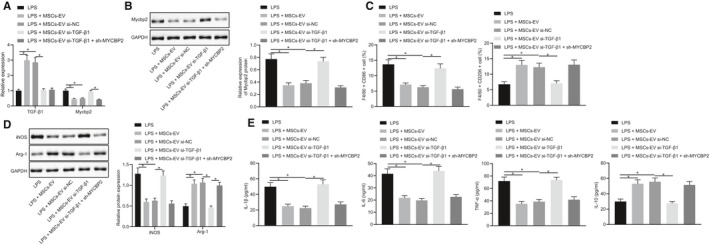
MSCs‐EVs carrying TGF‐β1 up‐regulate miR‐132 expression and thus inhibit MYCBP2 expression, polarizing macrophages towards M2 phenotype in vitro. A, mRNA expression of Mycbp2 and TGF‐β1 was determined by RT‐qPCR in the co‐culture system of RAW264.7 cells with MSCs‐EVs treated with si‐TGF‐β1 or in combination with sh‐Mycbp2, normalized to GAPDH. B, Representative Western blots of Mycbp2 protein and its quantitation in the co‐culture system of RAW264.7 cells with MSCs‐EVs treated with si‐TGF‐β1 or in combination with sh‐Mycbp2, normalized to GAPDH. C, Quantitative analysis of cell ratio of CD86^+^ and CD206^+^ in F4/80 cells detected by flow cytometry in the co‐culture system of RAW264.7 cells with MSCs‐EVs treated with si‐TGF‐β1 or in combination with sh‐Mycbp2. D, Representative Western blots of iNOS and Arg‐1 proteins and their quantitation in the co‐culture system of RAW264.7 cells with MSCs‐EVs treated with si‐TGF‐β1 or in combination with sh‐Mycbp2, normalized to GAPDH. E, Expression of inflammatory factors was measured by ELISA in cell supernatant in the co‐culture system of RAW264.7 cells with MSCs‐EVs treated with si‐TGF‐β1 or in combination with sh‐Mycbp2. The data were conducted by one‐way ANOVA with Tukey's post hoc test. Data are shown as mean ± SD of three technical replicates. *, *P* value between groups was less than 0.05

The aforementioned results had revealed that MSCs‐EVs could carry TGF‐β1 to promote the expression of miR‐132 and thus promote M2 polarization, and that miR‐132 could target Mycbp2. In order to further study the effect of TGF‐β1 carried by MSCs‐EVs on the regulation of Mycbp2 in polarization of macrophages via miR‐132, we co‐cultured the RAW264.7 cells with MSCs‐EVs treated with si‐TGF‐β1 or in combination with sh‐Mycbp2. The mRNA expression of TGF‐β1 and Mycbp2 was determined by RT‐qPCR and Mycbp2 protein expression by Western blot analysis. As depicted in Figure 6A,B, TGF‐β1 expression was elevated, while Mycbp2 expression was down‐regulated in cells co‐cultured with MSCs‐EVs or MSCs‐EVs treated with si‐NC. By contrast, these effects were reversed in cells co‐cultured with MSCs‐EVs treated with si‐TGF‐β1. Furthermore, the expression of TGF‐β1 and Mycbp2 presented with a decline in cells in the presence of concomitant silencing of TGF‐β1 and Mycbp2.

Flow cytometry was subsequently used to detect the M1 macrophage surface marker CD86 and M2 macrophage surface marker CD206. The results (Figure 6C) displayed that CD86^+^ cells were reduced, while CD206^+^ cells were increased in cells co‐cultured with MSCs‐EVs or MSCs‐EVs treated with si‐NC, which was counteracted by TGF‐β1 silencing. No alterations were observed in the CD86^+^ and CD206^+^ cells in RAW264.7 cells co‐cultured with MSCs‐EVs treated with si‐NC or with concomitant silencing of TGF‐β1 and Mycbp2. The results of Western blot analysis demonstrated that iNOS expression was attenuated, while Arg‐1 expression was augmented in RAW264.7 cells co‐cultured with MSCs‐EVs or MSCs‐EVs treated with si‐NC, which was rescued by TGF‐β1 silencing. Likewise, there were no changes regarding the iNOS and Arg‐1 expressions in RAW264.7 cells co‐cultured with MSCs‐EVs treated with si‐NC or with both si‐TGF‐β1 and sh‐Mycbp2 (Figure 6D). Cell supernatant exhibited decreased IL‐1β, IL‐6 and TNF‐α levels while elevated IL‐10 levels in RAW264.7 cells in response to co‐culture with MSCs‐EVs or MSCs‐EVs treated with si‐NC. On the contrary, silencing of TGF‐β1 could reverse the aforementioned tendency. In addition, no difference was found in RAW264.7 cells in response to co‐culture with MSCs‐EVs treated with si‐NC or both si‐TGF‐β1 and sh‐MYCBP2 (Figure 6E). The above data indicate that MSC‐derived EVs carrying TGF‐β1 elevate miR‐132 expression and down‐regulate MYCBP2 expression, consequently promoting M2 polarization of macrophages in vitro.

### 3.7 Mycbp2 enhances ubiquitination and degradation of TSC2 in macrophages

**FIGURE 7:**
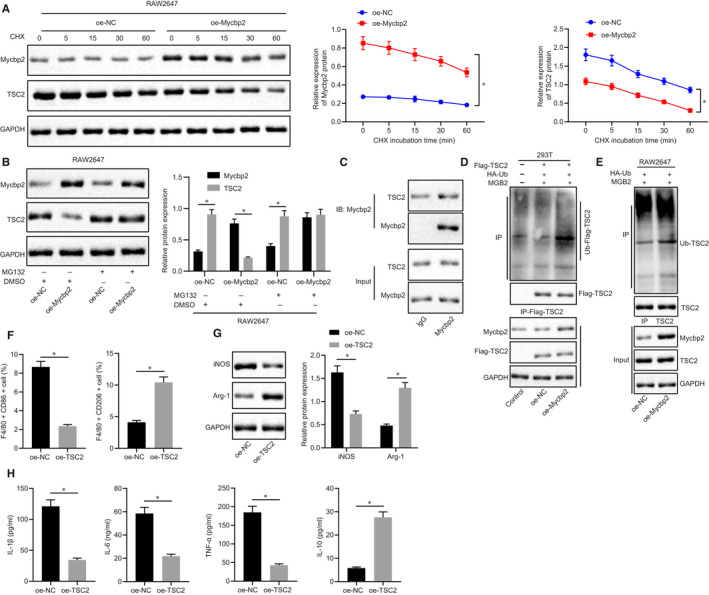
Mycbp2 ubiquitinates and degrades TSC2 in macrophages. A, Representative Western blots of Mycbp2 and TSC2 proteins and their quantitation in RAW264.7 cells treated with oe‐Mycbp2, normalized to GAPDH. B, Representative Western blots of Mycbp2 and TSC2 proteins and their quantitation in RAW264.7 cells treated with oe‐Mycbp2, MG132 or both, normalized to GAPDH. C, Co‐IP detection of the interaction between Mycbp2 and TSC2 in RAW264.7 cells. D, Effect of Mycbp2 on ubiquitination of TSC2 protein in 293T cells. E, Effect of Mycbp2 on ubiquitination of TSC2 protein in RAW264.7 cells. F, Quantitative analysis of cell ratio of CD86^+^ and CD206^+^ in F4/80 cells by flow cytometry in oe‐TSC2‐treated RAW264.7 cells. G, Representative Western blots of iNOS and Arg‐1 proteins and their quantitation in oe‐TSC2‐treated RAW264.7 cells. H, Expression of inflammatory factors was measured by ELISA in cell supernatant upon oe‐TSC2 treatment. The comparison between two groups was analysed using unpaired *t* test, while comparisons among multiple groups were conducted by one‐way ANOVA with Tukey's post hoc test. The data at different time‐points were compared by two‐way ANOVA followed by Dunnett's correction. Data are shown as mean ± SD of three technical replicates. *, *P* value between groups was less than 0.05

Mycbp2 has been reported to be able to ubiquitinate and degrade TSC2 protein,^15^ and TSC2 protein can promote M2 polarization of macrophages.^16^ In order to further study whether the stability of TSC2 protein was regulated by Mycbp2, we treated RAW264.7 cells with 100 μmol/L cycloheximide (CHX) and then determined the expression of TSC2 and Mycbp2 by Western blot analysis. Following treatment at the same time‐point, TSC2 protein expression was decreased in cells with oe‐Mycbp2, indicating that overexpression of Mycbp2 can reduce TSC2 stability (Figure 7A). Next, for verifying how Mycbp2 inhibited the stability of TSC2, we used MG132 to treat cells in each group and then determined the expression of Mycbp2 and TSC2 by RT‐qPCR. The results indicated enhanced Mycbp2 expression and decreased TSC2 expression in cells with oe‐Mycbp2. However, TSC2 expression was decreased in cells upon treatment of MG132, and Mycbp2 expression was elevated in cells with oe‐Mycbp2 and MG132 (Figure 7B). This result indicates that Mycbp2 can promote the degradation of TSC2 protein. As presented in Figure 7C, we found that TSC2 could interact with Mycbp2. Subsequently, with the aim to detect whether Mycbp2 promoted TSC2 protein degradation by promoting TSC2 ubiquitination, 293T cells were overexpressed Mycbp2 and cotransfected with HA‐Ub and Flag‐TSC2 and treated with 40 μmol/L MG132 for 48 hours. The findings revealed that (Figure 7D) the ubiquitination of TSC2 was increased in 293T cells overexpressing Mycbp2. Next, RAW264.7 cells were overexpressed Mycbp2 and cotransfected with HA‐Ub and treated with 40 μmol/L MG132 for 48 hours. The findings revealed that (Figure 7E) the ubiquitination of TSC2 was increased in RAW264.7 cells overexpressing Mycbp2. These results indicate that Mycbp2 can reduce the stability of TSC2, promote its degradation of ubiquitination‐proteasome, thereby inhibiting the expression of TSC2 protein level. To further study the effect of TSC2 on macrophage polarization, we knocked TSC2 down in RAW264.7 cells. There were decreased CD86^+^ cells, elevated CD206^+^ cells, reduced iNOS expression and enhanced Arg‐1 expression, as well as declined IL‐1β, IL‐6 and TNF‐α expressions while increased IL‐10 expression upon treatment of oe‐TSC2 (Figure 7F‐H). The aforementioned data supported that Mycbp2 advances ubiquitination and degradation of TSC2 in macrophages.

### 3.8 MSC‐secreted EVs carry TGF‐β1 to promote M2 polarization of macrophages via the miR‐132/Mycbp2/TSC2 axis in vitro

**FIGURE 8:**
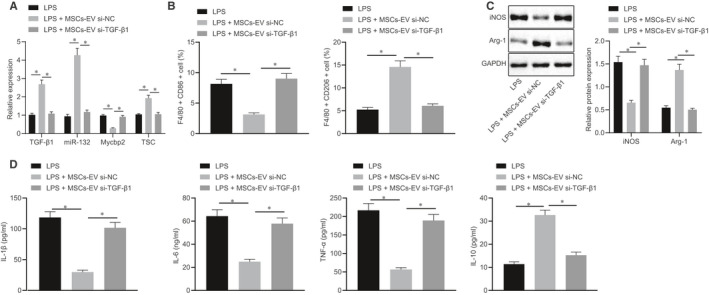
EVs from MSCs can carry TGF‐β1 to promote M2 polarization of macrophages through the miR‐132/Mycbp2/TSC2 axis. A, Expression of TGF‐β1, miR‐132, Mycbp2 and TSC2 was determined by RT‐qPCR in the co‐culture system of RAW264.7 cells with MSCs‐EVs treated with si‐TGF‐β1, normalized to U6 and GAPDH, respectively. B, Quantitative analysis of cell ratio of CD86^+^ and CD206^+^ in F4/80 cells by flow cytometry in the co‐culture system of RAW264.7 cells with MSCs‐EVs treated with si‐TGF‐β1. C, Representative Western blots of iNOS and Arg‐1 proteins and their quantitation in the co‐culture system of RAW264.7 cells with MSCs‐EVs treated with si‐TGF‐β1, normalized to GAPDH. D, Expression of inflammatory factors was measured by ELISA in cell supernatant in the co‐culture system of RAW264.7 cells with MSCs‐EVs treated with si‐TGF‐β1. The data of multiple groups were analysed by one‐way ANOVA with Tukey's post hoc test. Data are shown as mean ± SD of three technical replicates. *, *P* value between groups was less than 0.05

The next step was to further investigate the effect of MSCs‐EVs carrying TGF‐β1 on macrophage polarization. The findings indicated that TGF‐β1 and Mycbp2 expressions were reduced, while miR‐132 and Mycbp2 expressions were increased in RAW264.7 cells treated with LPS, co‐cultured with MSCs‐EVs or si‐TGF‐β1‐treated MSCs‐EVs (Figure 8A). Meanwhile, we also found that in RAW264.7 cells treated with LPS, co‐cultured with MSCs‐EVs or si‐TGF‐β1‐treated MSCs‐EVs, there were increased CD86^+^ cells, declined CD206^+^ cells, elevated iNOS expression and reduced Arg‐1 expression, as well as elevated IL‐1β, IL‐6 and TNF‐α expressions while decreased IL‐10 expression upon co‐culture with MSCs‐EVs treated with si‐TGF‐β1 (Figure 8B‐D). The above results suggest that EVs from MSCs can carry TGF‐β1 to promote M2 polarization of macrophages through the miR‐132/Mycbp2/TSC2 axis.

## 4 DISCUSSION

Evidence has shown that macrophages are able to differentiate into the anti‐inflammatory M2 phenotype or pro‐inflammatory M1 phenotype by influencing extracellular signalling within the tumour microenvironment.^23^ Tumour microenvironment–derived excessive TGF‐β has been suggested to block M1 macrophage development and also induce the activation of M2 macrophages.^24^ In the present study, we aimed to probe into the mechanisms of MSCs‐EVs carrying TGF‐β1 in M2 polarization of mouse macrophages. The obtained results indicate that MSCs‐EVs carrying TGF‐β1 can inhibit the Mycbp2‐TSC2 pathway by promoting miR‐132 expression, thereby promoting M2 polarization of mouse macrophages.

First, we found that MSCs promoted M2 polarization of LPS‐treated macrophages through EVs, and promoted the expression of IL‐10 and inhibited the expression of other inflammatory factors IL‐1β, TNF‐α and IL‐6. Several studies have demonstrated the functional interaction between MSCs and macrophages as well as the macrophage function by EVs derived from MSCs. For instance, MSCs have been found to promote M2 polarization and restrict M1 polarization in macrophages, which is widely considered as an essential stimulator in tissue regeneration.^25^ Human umbilical cord‐MSC‐treated macrophages presented an anti‐inflammatory phenotype with lower expressions of TNF‐α and IL‐1β, and higher expression of CD206.^26^ Moreover, another article has pointed out that bone marrow MSCs are capable of suppressing LPS‐induced M1 polarization and inducing to M2 polarization of macrophages through the production of paracrine factors.^27^ Meanwhile, the present study also revealed that MSC‐EV‐treated RAW264.7 cells had increased expression of TGF‐β1 that elevated the expression of miR‐132. Except for the roles on inflammatory responses, TGF‐β1 expression has been demonstrated to be up‐regulated in M1 macrophages, which is vital in cardiac fibroblast induction through mediating the TGF‐β/Smad signalling pathway.^28^ As reported, TGF‐β could stimulate macrophages and releasing anti‐inflammatory cytokine IL‐10, whereas LPS‐induced M1 macrophages secret pro‐inflammatory cytokines, such as TNF‐α and IL‐12.^24^ Although the role of TGF‐β1 in macrophage polarization has been extensively studied, it is still necessary to explore the function and downstream mechanistic basis of TGF‐β1 in EVs derived from MSCs. MSCs modified with TGF‐β1 gene can promote the polarization of M2 macrophages.^29^ Further, TGF‐β1 has been demonstrated to induce miR‐132 expression in multiple studies.^30^, ^31^ In addition, miR‐132 is capable of inducing M2 polarization in macrophages by targeting its specific transcription factor and adaptor protein.^32^ Additionally, the increased expression of miR‐132 has been found in murine macrophages cultured with ginseng stem‐leaf saponins (GSLS) and/or thimerosal (TS).^33^ miR‐132 expresses highly during the inflammatory phase of wound repair, and concurrently, TGF‐β1 can promote miR‐132 expression in keratinocytes.^13^ miR‐132 in human macrophages has been described as a regulator of the interferon‐γ‐induced macrophage activation pathway.^34^ Additionally, miR‐132 has been elucidated to exert anti‐inflammatory functions in alveolar macrophages, and LPS‐induced rat alveolar macrophages contributed to an enhancement in miR‐132 expression and high TNF‐α, IL‐1β and IL‐6 levels.^35^ Nevertheless, the presence of other mRNAs or miRNAs in EVs cannot be ruled out, which may play a role in cell crosstalk. This needs to be further investigated in future studies.

In order to further elucidate the downstream mechanism of miR‐132 in regulating macrophage differentiation, we found that miR‐132 could target Mycbp2. Mycbp2 is an inhibitor of the M2 macrophage polarization, which executes its suppressive effect via various signalling pathway.^36^ In addition to that, we also found that overexpression of Mycbp2 reversed the effects of overexpressed miR‐132 on M2 polarization of macrophages, indicating that miR‐132 can target Mycbp2 in macrophages to promote M2 polarization of macrophages. Mycbp2 can act as an E3 ubiquitin ligase towards tuberin and modulate mTOR signalling, implying that Mycbp2 in turn controls cell growth and neuronal function via the TSC/mTOR pathway in mammalian cells.^15^ Translation processes are modulated by Mycbp2 mainly through the mTOR signalling where Mycbp2 has a double function by ubiquitylating the Rheb inhibitor TSC2.^37^ As reported, TSC1/2 complex inhibits the Ras GTPase pathway to restrict M1 response, and its vital role in M2 activation is basically regulated by suppressing the mTOR pathway.^38^ Finally, we found that MSC‐secreted EVs carried TGF‐β1 to promote M2 polarization of macrophages via the miR‐132/Mycbp2/TSC2 axis. Similar to our study, a recent article has revealed that MSCs‐EVs treatment decreased the atherosclerotic plaque and greatly attenuating the infiltration of macrophages, thereby inducing M2 polarization of macrophages through the miR‐let7/IGF2BP1/PTEN pathway.^39^

**FIGURE 9:**
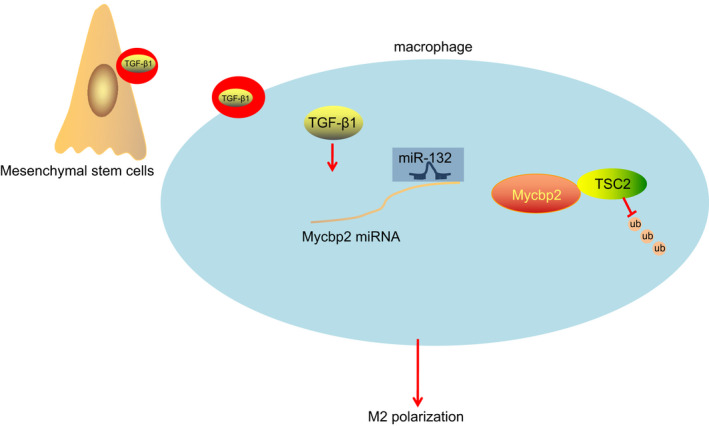
The mechanistic diagram depicts that MSC‐derived EVs carrying TGF‐β1 promote macrophage M2 polarization through the miR‐132/Mycbp2/TSC2 axis. In brief, MSC‐derived EVs carry TGF‐β1 into macrophages, promote the expression of miR‐132 and then inhibit the expression of Mycbp2 which promotes ubiquitination and degradation of TSC2 protein, ultimately polarizing macrophages towards M2 phenotype

In conclusion, the current study highlights that EVs derived from MSCs carrying TGF‐β1 promote M2 polarization of mouse macrophages through regulating the miR‐132/Mycbp2/TSC2 axis (Figure 9). These findings may provide a molecular basis for the application of MSCs‐EVs as an agent for macrophage M2 polarization. Additionally, the identification of the regulatory role of the miR‐132/Mycbp2/TSC2 axis in macrophage activation provides potent targets for macrophage‐associated diagnostic and therapeutic strategies.

## CONFLICT OF INTEREST

The authors declare that there is no conflict of interest associated with the manuscript.

## AUTHOR CONTRIBUTIONS

**Yongqi Wang:** Data curation (equal); Investigation (equal); Project administration (equal); Resources (equal); Supervision (equal); Writing‐original draft (equal). **Biao Han:** Conceptualization (equal); Investigation (equal); Methodology (equal); Writing‐original draft (equal). **Yingbin Wang:** Formal analysis (equal); Resources (equal); Validation (equal); Writing‐review & editing (equal). **Chunai Wang:** Formal analysis (equal); Software (equal); Validation (equal); Writing‐review & editing (equal). **Hong Zhang:** Data curation (equal); Investigation (equal); Writing‐review & editing (equal). **Jianjun Xue:** Formal analysis (equal); Visualization (equal); Writing‐review & editing (equal). **Xiaoqing Wang:** Data curation (equal); Investigation (equal); Writing‐review & editing (equal). **Tingting Niu:** Conceptualization (equal); Writing‐review & editing (equal). **Zhen Niu:** Conceptualization (equal); Writing‐review & editing (equal). **Yuhe Chen:** Conceptualization (equal); Writing‐review & editing (equal).

## ETHIC APPROVAL

All the animals’ experiments were performed at the Animal Center of the First Hospital of Lanzhou University and approved by the Animal Experiment Ethics Committee of the First Hospital of Lanzhou University.

## Supporting information

Figure S1

## Data Availability

The data used to support the findings of this study are available from the corresponding author upon request.
